# Estimation of antimicrobial resistance of *Mycoplasma genitalium,* Belgium, 2022

**DOI:** 10.2807/1560-7917.ES.2024.29.7.2300318

**Published:** 2024-02-15

**Authors:** Irith De Baetselier, Hilde Smet, Kaat Kehoe, Imelda Loosen, Marijke Reynders, Iqbal Mansoor, Lorenzo Filippin, Mathieu Cauchie, Ellen Van Even, Nadia Makki, Gilberte Schiettekatte, Wouter Vandewal, Bart Glibert, Veerle Matheeussen, Yolien Van der Beken, Reinoud Cartuyvels, Sophia Steyaert, Ann Lemmens, Maria-Grazia Garrino, Henry Paridaens, Elena Lazarova, Bénédicte Lissoir, Marine Deffontaine, Amélie Heinrichs, Veroniek Saegeman, Elizaveta Padalko, Amaryl Lecompte, Wim Vanden Berghe, Chris Kenyon, Dorien Van den Bossche

**Affiliations:** 1 National Reference Centre of Sexually Transmitted Infections Belgium, Institute of Tropical Medicine, Department of Clinical Sciences, Antwerp, Belgium; 2 Algemeen Medisch Laboratorium, Antwerp, Belgium; 3 Laboratoire Bauduin, Enghien, Belgium; 4 AZ (General Hospital) Sint-Jan Brugge-Oostende AV, Laboratory Medicine, Molecular Microbiology, Bruges, Belgium; 5 Labo Epicura, Ath, Belgium; 6 Europa Ziekenhuizen, Brussels, Belgium; 7 Clinical Laboratory of Microbiology, Heilig Hart (HH) Hospital Lier, Lier, Belgium; 8 Centrum voor Medische Analyse, Herentals, Belgium; 9 AZ Sint-Lucas, Bruges, Belgium; 10 AZ Glorieux, Ronse, Belgium; 11 Department of Microbiology, University Hospital Antwerp, Edegem, Belgium; 12 Klinisch Laboratorium Maenhout Waregem, Waregem, Belgium; 13 Clinical Laboratory, Jessa Hospital, Hasselt, Belgium; 14 AZ Maria Middelares, Gent, Belgium; 15 AZ Sint-Maarten Hospital, Department Clinical Microbiology, Mechelen, Belgium; 16 Centre Hospitalier Régional de Namur, site Meuse, Namur, Belgium; 17 Laboratory of Molecular Biology, Citadelle Hospital, Liege, Belgium; 18 Clinical laboratory, Regional Hospital de la Haute Senne, Soignies, Belgium; 19 Grand Hôpital De Charleroi Saint-Joseph, Gilly, Belgium; 20 Laboratoire de Biologie Clinique, Centre Hospitalier de Mouscron, Mouscron, Belgium; 21 Laboratory of Clinical Biology, Hospital Arlon, Vivalia, Arlon, Belgium; 22 Clinical Laboratory, Vitaz hospital, Sint-Niklaas, Belgium; 23 Ghent University Hospital, Department of Laboratory Medicine, Ghent, Belgium; 24 Department of Epidemiology and Public Health, Sciensano, Brussels, Belgium; 25 STI Unit, Department of Clinical Sciences, Institute of Tropical Medicine, Antwerp, Belgium

**Keywords:** *Mycoplasma genitalium*, antimicrobial resistance, Europe, Belgium, fluoroquinolone resistance, macrolide resistance

## Abstract

**Background:**

Antimicrobial resistance (AMR) of *Mycoplasma genitalium* (MG) is a growing concern worldwide and surveillance is needed. In Belgium, samples are sent to the National Reference Centre of Sexually Transmitted Infections (NRC-STI) on a voluntary basis and representative or robust national AMR data are lacking.

**Aim:**

We aimed to estimate the occurrence of resistant MG in Belgium.

**Methods:**

Between July and November 2022, frozen remnants of MG-positive samples from 21 Belgian laboratories were analysed at the NRC-STI. Macrolide and fluoroquinolone resistance-associated mutations (RAMs) were assessed using Sanger sequencing of the 23SrRNA and *parC* gene. Differences in resistance patterns were correlated with surveillance methodology, socio-demographic and behavioural variables via Fisher’s exact test and logistic regression analysis.

**Results:**

Of the 244 MG-positive samples received, 232 could be sequenced for macrolide and fluoroquinolone RAMs. Over half of the sequenced samples (55.2%) were resistant to macrolides. All sequenced samples from men who have sex with men (MSM) (24/24) were macrolide-resistant. Fluoroquinolone RAMs were found in 25.9% of the samples and occurrence did not differ between socio-demographic and sexual behaviour characteristics.

**Conclusion:**

Although limited in sample size, our data suggest no additional benefit of testing MG retrieved from MSM for macrolide resistance in Belgium, when making treatment decisions. The lower occurrence of macrolide resistance in other population groups, combined with emergence of fluoroquinolone RAMs support macrolide-resistance testing in these groups. Continued surveillance of resistance in MG in different population groups will be crucial to confirm our findings and to guide national testing and treatment strategies.

**Key public health message**

**What did you want to address in this study and why?**

*Mycoplasma genitalium* (MG) is a bacterium causing sexually transmitted infections manifesting as urethritis and cervicitis which are commonly treated with macrolides or fluoroquinolones. However, antimicrobial resistance (AMR) of MG is becoming a global health issue and multidrug resistance is increasing. To correctly manage MG infections, macrolide resistance testing is recommended. We performed a survey to estimate AMR in MG in Belgium.

**What have we learnt from this study?**

More than half of the MG-positive samples harboured resistance-associated mutations (RAMs) to macrolides, the primary choice for treatment of MG. All samples from men who have sex with men (MSM) were macrolide-resistant. This was much less in women (45%). The presence of fluoroquinolone RAMs was around 26% and did not differ between different populations.

**What are the implications of your findings for public health?**

The study results suggest that testing for macrolide-resistance among MSM might not be needed in Belgium, but it is crucial in other population groups due to the lower prevalence of macrolide resistance, combined with the emerging fluoroquinolone resistance. Continued surveillance is vital to guide national testing and treatment strategies to effectively manage AMR in MG infections.

## Introduction

*Mycoplasma genitalium* (MG) causes sexually transmitted infections (STI) manifesting as urethritis and cervicitis. In high-income countries, the prevalence of MG is estimated to 1.3% without significant differences between men and women [1]. In Belgium, previous studies have reported higher prevalences, but these were estimated from populations behaviourally more at risk, such as men who have sex with men (MSM) (17.2%) and females who engage in sex work (10.8%) [2,3]. In a recent Dutch study, a prevalence of 13.8% was estimated among individuals visiting an STI clinic: 8.2% among men who have sex with women (MSW), 12.6% in women and 20.1% among MSM [4]. These figures were higher than previously reported.

Although MG is considered prevalent in sexually active individuals, the majority of infections are asymptomatic and can clear spontaneously. Data on the potential consequences of untreated asymptomatic infections are limited, making it difficult to support widespread MG screening [5,6]. Moreover, the ability of MG to acquire resistance to azithromycin and moxifloxacin, which are currently the most prescribed antimicrobials to treat MG infections, is of concern [7]. Doxycycline, the recommended first-line therapy for non-gonococcal urethritis in Europe and the United States (US), has a low cure rate of 30–40% and is now predominantly used in two-stage therapy approaches for MG [6,8,9]. These approaches use doxycycline first to lower the bacterial load of MG and afterwards, a second antimicrobial (azithromycin or moxifloxacin) is administered according to the susceptibility profile of the pathogen [5].

In Belgium, we previously reported a high macrolide resistance of 68.3% in MG [10]. In addition, 18.0% of the samples also harboured resistance-associated mutations (RAMs) to fluoroquinolones. However, these numbers may not accurately reflect the resistance in the population since most samples were from MSM (95/167; 56.9%).

Since 2015, the National Reference Centre of Sexually Transmitted Infections (NRC-STI) in Belgium has included the confirmation of MG in surveillance. However, since 2018, a strict testing strategy has been applied to follow the guidelines of the International Union against STIs (IUSTI, https://iusti.org/). This testing strategy entails that testing is performed, in most cases, only in the event of persistent urethritis or cervicitis and when chlamydia or gonorrhoea have been excluded [7]. As a consequence, the yearly number of samples tested is low and may not be representative of the entire Belgian population.

Other countries, such as France and the United Kingdom (UK) have performed additional surveys to estimate antimicrobial resistance (AMR) of MG in samples from sexual health clinics or laboratories during a 1–3-month period [11-13]. These kinds of surveys may provide a more comprehensive picture of MG AMR. In 2020, the presence of macrolide RAMs was estimated at 60.2% in men and 22.2% in women in France. Fluoroquinolone RAMs were present in 17.3% and in 15.2% of the male and female samples, respectively, which was lower than the estimates in Belgium [11].

The aim of this study was to perform a similar survey in Belgium to estimate the AMR of MG. Moreover, we collected socio-demographic, behavioural and clinical data from individuals with MG-positive samples.

## Methods

### National surveillance of *Mycoplasma genitalium* in Belgium

In Belgium, laboratories can send samples suspected for MG infection for analysis to the NRC-STI only when the request is in accordance with the IUSTI 2021 guidelines [7].

The extraction of DNA is performed using the Abbott m2000sp instrument (Abbott Molecular, Des Plaines, United States (US)) and *Chlamydia trachomatis* (CT)/*Neisseria gonorrhoeae* (NG) extraction kit according to manufacturer’s instructions. Detection of MG is performed using the S-DIaMGTV multiplex kit (Diagenode Diagnostics, Seraing, Belgium) using the Abbott m2000rt platform. Left-over DNA extracts are stored at below −20°C for further testing.

### Molecular detection of macrolide and fluoroquinolone resistance-associated mutations

Specific in-house PCRs were used to retrieve amplicons of 23S rRNA and the partitioning protein C (*parC*) gene using the GeneAmp PCR System 2700 (Applied Biosystems, Waltham, US) on the left-over DNA extracts, if available [14,15]. The extraction of DNA was performed using the same methodology as mentioned above if not already performed at the NRC-STI. At the Neuromics Support Facility – VIB, Antwerp, Belgium, Sanger sequencing of the 23S rRNA and *parC* genes to detect the presence of RAMs to macrolides and fluoroquinolones respectively was performed using the ABI 36730xl instrument of Applied Biosystems as previously described [10]. Sequences were aligned and analysed by two independent readers using BioEdit Sequence Alignment Editor (nucleics.com). The following alterations in ParC were coded to be resistant to fluoroquinolones: S83I, S83R, D87N and D87Y.

### Design of a prospective survey on antimicrobial resistance of *Mycoplasma genitalium*

All Belgian laboratories were contacted and asked in June 2022 to complete a questionnaire on MG testing and their willingness to participate in a prospective study beginning in July 2022. The survey included questions on the molecular testing technique, restriction rules applied to MG testing, if applicable, and the number of samples tested between 1 January and 31 March 2022 (Q1), including positivity ratio. The participating laboratories were asked to send a convenience sample of ca 12, maximum 20, frozen remnants of consecutively collected MG-positive samples to the NRC-STI between July and November 2022. An additional survey with socio-demographic and sexual behaviour questions was completed for each positive sample. On arrival at the NRC-STI, samples were thawed and tested for the presence of macrolide and fluoroquinolone RAMs as described above.

Laboratories analysing over 1,000 samples in Q1 2022 were asked to provide MG positivity ratios for 2022.

### Statistical methods

A descriptive analysis was performed for all MG cases. In case of returning visits to a physician, only results from individuals with an interval of > 90 days were included in the analysis. Resistance patterns (macrolides, fluoroquinolones or both) were correlated with sampling method, geographical location, biological sex, transmission and the presence of symptoms by using Fisher’s exact test. If statistical significance of p < 0.05 was calculated, this variable was included in a multivariate logistic regression model. Sequenced samples of MG with unknown variables were excluded from the multivariate logistic regression model. An adjusted odds ratio (aOR) with the corresponding 95% confidence interval (CI) was reported. We used STATA V15.1 (StataCorp LP, College Station, Texas, US) for all statistical analyses. A significance level of 0.05 was applied.

## Results

### Testing strategy of *Mycoplasma genitalium* in Belgium

A total of 21 hospital and private laboratories participated in the study and all three regions of Belgium were represented. The exact location and more details of the laboratories, including the number of samples tested during Q1 2022, the number of positive samples, the molecular technique of MG detection and the number of samples shipped to the NRC-STI for further AMR testing can be seen in Supplementary Figure S1 and Supplementary Table S2. Testing was mostly performed in the framework of a broader STI screening by performing a multiplex assay which included at minimum *C. trachomatis*, *N. gonorrhoeae*, *M. genitalium* and *Trichomonas vaginalis* (16/21 laboratories) or at the clinician’s request. No testing restrictions were imposed by any of the laboratories. Positivity ratio of MG in Q1 2022 varied between 0 and 14.0%.

### Findings of *Mycoplasma genitalium*

In 2022, the NRC-STI received 351 MG suspected samples for MG testing. A total of 29.1% (102/351) of the requests did not comply with the testing guidelines and were discarded. Of the remaining 249 samples, 36 (14.5%) were positive for MG. The MG positivity ratios, stratified by sex, obtained by the NRC-STI and by the four laboratories that tested over 1,000 samples in Q1 2022 are shown in Table 1.

**Table 1:** Detection of *Mycoplasma genitalium*, by region and sex, Belgium, 2022

Laboratory identification	Region	All tested			Men			Women		
		n	Positive	%	n	Positive	%	n	Positive	%
NRC-STI^a^	Flanders	249	36	14.5	180	27	15.0	69	9	13.0
Private laboratory 1^b^	Flanders	35,927	287	0.8	12,265	91	0.7	23,662	196	0.8
Private laboratory 2^c^	Wallonia	2,527	121	4.8	260	16	6.2	2,267	105	4.6
Hospital laboratory^d^	Flanders	5,247	267	5.1	1,413	172	12.2	3,834	95	2.5
Private laboratory 3^c^	Wallonia	8,385	401	4.8	Not available			Not available		

NRC-STI: National Reference Centre of Sexually Transmitted Infections in Belgium; CT/NG: *Chlamydia trachomatis/Neisseria gonorrhoeae*; MG: *Mycoplasma genitalium*; STI: sexually transmitted infections.

^a^ Only samples of symptomatic individuals, from which CT/NG was excluded, were tested.

^b^ Multiplex testing: samples were tested for CT/NG/MG without any restriction. Results for MG were provided when CT/NG was negative.

^c^ Multiplex testing: samples were tested for CT/NG/MG without any restriction.

^d^ Multiplex testing: samples from symptomatic individuals (STI clinics) and of asymptomatic individuals from risk populations (including young adults < 25 years and pregnancy screening of risk populations).

### Demographics and behavioural characteristics of individuals with samples positive for *Mycoplasma genitalium*

Besides the 36 samples detected by the NRC-STI, 208 samples were collected within the prospective study, resulting in 244 positive samples. The origin of the samples and socio-demographic and behavioural characteristics and symptoms of the persons from whom these samples were obtained are summarised in Table 2. Almost all samples were of urogenital origin (229/244; 93.9%) and more than half were obtained from women (154/244; 63.1%). A gynaecologist took 38.1% (93/244) of the samples, followed by a general practitioner (80/244; 32.8%) and 30 (12.3%) samples were taken by other clinicians such as urologists, infectious disease specialists, dermatologists, surgeons or emergency doctors. Of the cases with information about the presence or absence of the symptoms, 74.1% (137/185) presented with symptoms and 48 were asymptomatic. Notably, MG was also present in patients with nonspecific symptoms such as vaginal pain, blood loss, genital irritation or itching, burning sensation, discharge and urogenital or abdominal pain (58/185; 31.4%). Tests of most of the asymptomatic cases were requested by gynaecologists (26/48) and general practitioners (14/48).

**Table 2:** Socio-demographic and behavioural characteristics of cases with samples positive for *Mycoplasma genitalium,* Belgium, 2022 (n = 244)

Characteristics	Number	%
Median age (IQR)	28.7 years (23.1–36.9)	
Source		
Surveillance (NRC-STI)	36	14.8
Prospective study	208	85.2
Biological sex		
Female	154	63.1
Male	87	35.7
Unknown	3	1.2
Region of residence		
Brussels	49	20.1
Flanders	123	50.4
Wallonia	72	29.5
Sampling physician		
Gynaecologist	93	38.1
General practitioner	80	32.8
STI clinician	7	2.9
Other	30	12.3
Unknown	34	13.9
Transmission		
Heterosexual (including all women)	185	75.8
Men who have sex with men	24	9.8
Bisexual men	1	0.4
Unknown	34	13.9
Specimen type		
Genital secretion	147	60.2
Urine	82	33.6
Pooled sample (anorectal, pharyngeal and urine)	6	2.5
Anorectal	2	0.8
Unknown	7	2.9
Clinical diagnosis or symptoms		
Urethritis	48	19.7
Cervicitis	19	7.8
PID	8	3.3
Lesions	3	1.2
Proctitis	1	0.4
Nonspecific urogenital symptoms	58	23.8
Vaginal blood loss	24	9.8
Other genital discharge	8	3.3
Genital irritation or itching	5	2.0
Abdominal pain	11	4.5
Urogenital pain^a^	10	4.1
Asymptomatic or unknown symptoms		
No symptoms	48	19.7
Unknown	59	24.2
CT or NG co-infection		
Yes	21	8.6
No	206	84.4
Unknown	17	7.0

CT: *Chlamydia trachomatis*; IQR: interquartile range; NG: *Neisseria gonorrhoeae*; NRC-STI: National Reference Centre of Sexually Transmitted Infections in Belgium; PID: pelvic inflammatory disease; STI: sexually transmitted infections.

^a^ Urogenital pain includes dysuria (n = 2), testicular pain (n = 3), vaginal pain (n = 4), genital pain not specified (n = 1).

### Resistance-associated mutations

**Figure 1:**
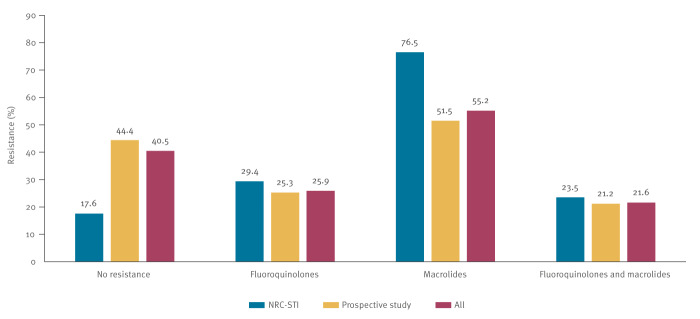
Percentage of resistance-associated mutations of *Mycoplasma genitalium* in samples from symptomatic and asymptomatic cases*,* Belgium, 2022 (n = 244)

Sequencing for both macrolide and fluoroquinolone RAMs was successful for 232 of 244 (95.1%) samples. Of these 232 samples, 34 (14.7%) were collected in the framework of the NRC-STI MG surveillance and 198 (85.3%) in the prospective study. Figure 1 depicts the antimicrobial resistance patterns of all samples stratified by collection method (NRC-STI or prospective study) and Table 3 shows the antimicrobial resistance patterns for the included characteristics.

NRC-STI: National Reference Centre of Sexually Transmitted Infections in Belgium.

**Table 3:** Antimicrobial resistance mutation patterns of *Mycoplasma genitalium,* by demographic and sexual risk behaviour, Belgium, 2022 (n = 232)

Characteristics	Total		Wild type				Macrolide resistance				Fluoroquinolone resistance^a^					Combined resistance					
	n	%	n	%	95% CI	p value	n	%	95% CI	p value	n	%	95% CI		p value	n	%	95% CI			p value
Origin of samples																					
NRC-STI	34	14.7	6			0.004	26			0.008	10				0.672	8					0.822
Prospective study	198	85.3	88	44.4	37.4–51.7		102	51.5	44.3–58.7		50	25.3	19.4–31.9			42	21.2			15.7–27.6	
Region of residence																					
Brussels	47	20.3	12			**0.037**	33			**0.025**	13				0.276	11					0.106
Flanders	116	50.0	48	41.4	32.3–50.9		64	55.2	45.7–64.4		34	29.3	21.2–38.5			30	25.9		18.2–34.8		
Wallonia	69	29.7	34	49.3	37.0–61.6		31	44.9	32.9–57.4		13	18.9	10.4–30.1			9	13.0	6.1–23.3			
Biological sex																					
Female	143	61.6	71	49.7	41.2–58.1	**< 0.001**	64	44.8	36.4–53.3	**< 0.001**	36	25.2	18.3–33.1		0.598	28	19.6	13.4–27.0			0.270
Male^b^	87	37.5	22	25.3	16.6–35.7		63	72.4	61.8–81.5		23	26.4	17.6–37.0			21	24.1	15.6–34.5			
Unknown	2	0.9	1				1				1					1					
Transmission																					
Women^c^	143	61.6	71	49.7	41.2–58.1	**< 0.001**	64	44.8	36.4–53.3	**< 0.001**	36	25.2		18.3–33.1	0.776	28	19.6		13.4–27.0		0.574
MSM^a^	24	10.3	0				24				6					6					
MSW	30	12.9	11				18				10					9					
MSU or unknown gender	35	15.1	12				22				8					7					
STI-associated symptoms																					
No	45	19.4	21			0.657	21			0.440	12				1.000	9					0.976
Yes	133	57.3	52	39.1	30.8–47.9		76	57.1	48.3–65.7		34	25.6		18.4–33.8		29	21.8		15.1–29.8		
Unknown	54	23.3	21	38.9			31	57.4	43.2–70.8		14	25.9		15.0–39.7		12	22.2		12.0–35.6		
CT or NG infection																					
No	196	84.5	82	41.8	34.8–49.1	0.815	106	54.1	46.8–61.2	1.000	49	25.0		19.1–31.7	0.788	41	20.9		15.4–27.3		1.000
Yes	20	8.6	9				11				4					4					
Unknown	16	6.9	3				11				7					5					
Total	232		94	40.5	34.1–47.1		128	55.2	48.5–61.7		60	25.9		20.4–32.0		50	21.6		16.4–27.4		

CI: confidence interval; CT: *Chlamydia trachomatis*; MSM: men who have sex with men; MSU: men with unknown sex of sex partners or unknown gender; MSW: men who have sex with women; NG: *Neisseria gonorrhoeae*; NRC-STI: National Reference Centre of Sexually Transmitted Infections in Belgium; ParC: partitioning protein C; STI: sexually transmitted infections.

^a^ Resistance-associated mutation leading to an alteration in ParC at position 83 or 87.

^b^ One transwoman included.

^c^ One woman reported same sex transmission.

Percentages not included where n < 60.

Macrolide resistance was detected in 128 (55.2%) of 232 samples, however, macrolide resistance was higher in samples collected in the framework of the NRC-STI surveillance; highest in men and in Brussels. All MG from samples collected from MSM harboured RAMs associated with macrolide resistance. Macrolide resistance was also higher in MSW (18/30) than in women (64/143; 44.8%).

Variation in fluoroquinolone resistance was small between the demographic groups: MSW had the highest proportion of fluoroquinolone RAMs (10/30) compared with MSM (6/24), but this was not statistically significant. In total, more than one in four of the samples had RAMs to fluoroquinolones and one in five had combined fluoroquinolone and macrolide RAMs. Besides the clear association between macrolide resistance and MSM, the multivariate logistic regression model, which included sex and region of residence, showed that macrolide resistance was less prevalent among women (aOR: 0.34; 95% CI: 0.19–0.61).

Table 4 tabulates the RAMs found in the study stratified by sexual transmission. Interestingly, A2058G was more frequently found among women and MSW, whereas A2059G was the most dominant mutation among MSM and men with unknown sex of partners or unknown gender (MSU) and D87N and S83I were the most frequently found alterations in ParC. Resistance-associated mutations to both antimicrobials were found in 50 samples and the following combinations were the most frequently found: A2058G/D87N (48.0%; 24/50) and A2059G/S83I (36.0%; 18/50).

**Table 4:** Resistance-associated mutations to macrolides (23S rRNA) and fluoroquinolones (alterations in ParC) in *Mycoplasma genitalium,* Belgium, 2022 (n = 138)^a^

Mutation	Total		Sex and transmission route			
	n	%	Women	MSM	MSW	MSU
23S rRNA (*Escherichia coli* numbering) (n = 128)						
A2058C	1	0.8	0	0	0	1
A2058G	49	38.3	32	5	8	4
A2058T	23	18.0	13	4	3	3^b^
A2059C	1	0.8	1	0	0	0
A2059G	54	42.2	18	15	7	14
Alterations in ParC (n = 62)						
D87N	29	46.8	21	3	5	0
D87Y	3	4.8	3	0	0	0
S83I	27	43.6	12	3	5	7
S83R	1	1.6	0	0	0	1
S83C^c^	1	1.6	0	1	0	0
S83N^c^	1	1.6	1	0	0	0

MSM: men who have sex with men; MSW: men who have sex with women; MSU: men with unknown sex of sex partners or unknown gender; ParC: partitioning protein C; rRNA: ribosomal ribonucleic acid.

^a^ Samples with no mutations are not included.

^b^ Mixed infection of A2058G and A2058T.

^c^ Mutation was not considered as possible fluoroquinolone resistant.

Percentages not included where n < 60.

## Discussion

We discovered a considerable number of macrolide-resistant MG in different populations in Belgium. Half of the samples in this study were genotypically resistant to macrolides (55.2%). Although limited in sample size, all samples collected from MSM harboured RAMs to macrolides, which is probably associated with the use of macrolides to treat other STIs or other infections [16]. These treatment regimens (i.e. one dose of 1 g azithromycin) are sub-optimal for MG and may promote the development of AMR and persistence of macrolide resistant clones [7]. In 2022, treatment regimens for STIs were adapted in many countries, including Belgium, to limit the usage of azithromycin and subsequently, to prevent further emergence of azithromycin resistance in MG but also in other STI pathogens [17,18].

In our study, macrolide resistance in MG from women was 45%. Importantly, our study also identified differences in the distribution of RAMs among different populations. For example, RAM A2059G in 23SrRNA was the most dominant RAM found among MSM, while A2058G was most common among women and MSW. These differences suggest that the circulation of different MG genotypes may contribute to the spread of macrolide resistance in specific populations. Indeed, a previous phylogenetic analysis in Spain revealed that the spread of strains harbouring a A2058T mutation contributed to the increase in macrolide resistance [19]. Comparing our data with previously published data of 2018, we note an increase in the proportion of samples harbouring this A2058T mutation from 5.0% to 18.0% [10]. A similar increase was also observed in the Netherlands [20]. This finding highlights the need for additional genetic characterisation of MG to better understand the circulation of the genetic profiles of MG and possible clonal spread of macrolide resistant MG.

With the increasing availability of multiplex testing to screen for STIs in Belgium, new recommendations for MG testing and treatment are urgently needed. The positivity ratio of MG is dependent on the testing strategy used. At the NRC-STI, where only samples of symptomatic individuals are tested, MG positivity ratio was nearly 15.0%. However, lower positivity ratios were found in samples from laboratories testing MG in all samples collected for STI screening (0.8 to 5.0%). These estimates may reflect the actual prevalence of MG in the population and are consistent with estimates from other European countries [21,22].

It is essential to train physicians to test for MG only when necessary, considering that our study MG testing was performed even for asymptomatic cases. However, several diagnostic companies have developed multiplex assays detecting *C. trachomatis*, *N. gonorrhoeae*, *M. genitalium* and *T. vaginalis*. Incorporating MG into STI screening panels may result in excessive antimicrobial use, potentially fostering resistance as most laboratories will provide all the results to the physician [23]. Consequently, it might be advisable to consider restricting the use of such assays for STI screening if the result of unrequested organisms cannot be masked.

Importantly, our data could influence guidelines for testing and treatment. Despite the small sample size, we demonstrate that macrolide resistance testing may not be beneficial for MSM in Belgium. Nevertheless, it remains advisable to perform macrolide resistance testing in other populations. Importantly, larger studies are necessary to confirm these results before considering the restriction of macrolide resistance testing to specific groups.

In addition to macrolide resistance, we also observed an increasing prevalence of fluoroquinolone resistance among MG in our study. Specifically, we found that 25.9% of the MG isolates had RAMs to fluoroquinolones, with women showing a nearly threefold higher proportion in fluoroquinolone resistance in MG compared with 2018 (from 9.1 to 25.2%). Although the Belgian federal government implemented a reimbursement constraint on quinolones in May 2018, these agents continue to be extensively used in the treatment of uncomplicated urinary tract infections. This practice could potentially elucidate the surge in fluoroquinolone resistance observed in women [24,25]. However, although limited in sample size, we also observed that one in three of the MG samples of MSW harboured fluoroquinolone RAMs, but almost half of these strains were still susceptible to azithromycin. These data indicate that resistance-guided therapy should be implemented in specific population groups to ascertain that an appropriate antimicrobial is selected.

Unfortunately, in Belgium, sexual behaviour data are mostly not available to the laboratory to decide whether or not resistance testing is needed. Commercially available AMR assays exist and allow a more rapid turnaround time compared with sequencing, though these are expensive and mostly limited to detection of macrolide RAMs. Moreover, there is currently no reimbursement for MG testing nor for resistance testing, which in turn may be expensive for the patients. The NRC-STI is performing these tests as part of their reference tasks but with very strict testing rules.

Our results reinforce the need for revision of the MG testing guidelines and reimbursement rules aiming at MG testing only in case of persisting symptoms according to the IUSTI guidelines. Macrolide resistance testing of MG-positive samples should be implemented for patient groups other than MSM to limit the use of fluoroquinolones and thus, to avoid emergence of multidrug-resistant MG. One fifth of our samples presented with RAMs against both antimicrobials. As fluoroquinolone RAMs are not always associated with therapeutic failure, some patients can still successfully be treated with moxifloxacin [26]. However, clinical multidrug-resistant MG infections are increasingly being reported, also in Belgium [6]. Third-line therapies include minocycline, pristinamycin and chloramphenicol [7,27,28]. However, the latter two are difficult to procure in Belgium and novel treatment options should be investigated.

As such, there are limited treatment options for MG infections and monitoring of antimicrobial resistance to the most frequently used antimicrobials is crucial to tailor treatment and testing guidelines. Here, we showed that the current surveillance method for MG AMR in Belgium is sub-optimal and should be adjusted. As in France and the UK, a prospective survey annually or every other year, could provide a better estimation of the of MG AMR.

Our study had several limitations. Firstly, the laboratories did not use the same testing strategies nor did they all have access to socio-demographic and behavioural data. The MG positivity ratios only provide rough estimates of prevalence as they are impacted by testing strategies applied in the different laboratories. The variation in positivity ratios reflect the sampling bias and they cannot be used as an accurate estimation of the MG prevalence in Belgium. Secondly, our pragmatic testing strategy involved selecting a convenience sample size of ca 12 MG-positive samples per laboratory, collected between July and November 2022. However, it is important to acknowledge that certain laboratories contributed either more or fewer MG-positive samples potentially skewing the representativeness of the Belgian population. For example, the Flemish region was overrepresented in this study and our sample size of MSM and MSW was limited. Thirdly, we did not include *gyrA* sequencing. While the clinical significance of *gyrA* mutations is considered less crucial than *parC* mutations, recent data indicate that MG samples harbouring dual ParC S83I and GyrA M95/D99 alterations exhibit lower microbiological cure rates compared with MG samples that only have ParC S83I mutations [29]. This underscores the potential importance of including *gyrA* mutations in future surveillance programmes. Finally, we did not have any information about the administered treatment and subsequent clinical response rates and the presence of RAMs does not always translate to clinical resistance. Furthermore, it is important to note that test-of-cure for MG is not a routine procedure in Belgium. As such, our resistance figures may be overestimated. In a forthcoming national surveillance study, we will attempt to mitigate these limitations.

## Conclusion

Our study underscores the importance of accurate surveillance of antimicrobial resistance of MG to adapt testing- and treatment guidelines. Macrolide resistance testing is expensive and currently not reimbursed in Belgium. Although limited in sample size, our findings suggest the potential clinical irrelevance of this test within the MSM population. Nonetheless, this finding should be confirmed in larger surveys. Finally, we advocate for the prompt adoption of macrolide resistance testing for MG in other populations. Our findings highlight the requirement for updated Belgian MG testing and treatment guidelines, including comprehensive training for both laboratory specialists and healthcare practitioners. These measures are imperative in preventing testing practices that may inadvertently contribute to the further emergence of multidrug-resistant MG.

## Supplementary Data

310000 Supplementary Material
