# Supplementary material for: Estimation of antimicrobial resistance of Mycoplasma genitalium, Belgium, 2022
Source: Euro Surveill. 2024 Feb 15;29(7):2300318. doi: 10.2807/1560-7917.ES.2024.29.7.2300318 (PMC10986661; doi:10.2807/1560-7917.ES.2024.29.7.2300318)
Supplement: Supplementary Material [file 23-00318_BAETSELIER_Supplement.pdf]

This supplementary material is hosted by Eurosurveillance as supporting information alongside the article “Estimation of AMR of MG, Belgium, 2022, on behalf of the authors, who remain responsible for the accuracy and appropriateness of the content. The same standards for ethics, copyright, attributions and permissions as for the article apply. Supplements are not edited by Eurosurveillance and the journal is not responsible for the maintenance of any links or email addresses provided therein.

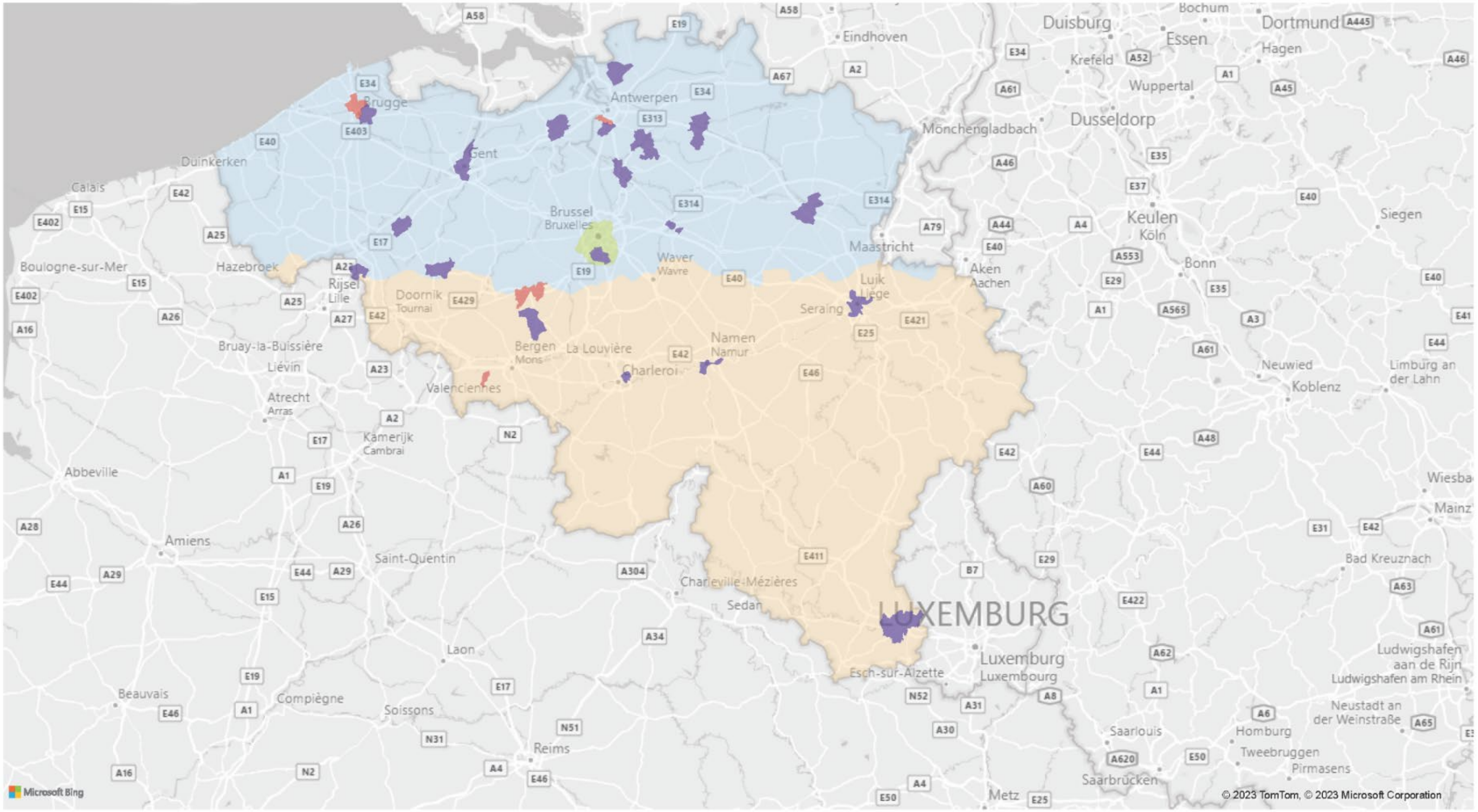

Supplementary Figure S1: Geographical location of laboratories participating in the prospective study. The red laboratories provided information about the prevalence of M. genitalium. The regions of Belgium are depicted in the following colours: Light blue: Flanders, Green: Brussels and Orange: Wallonia

Supplementary table S2: Mycoplasma genitalium testing characteristics including positivity ratio of Belgian laboratories who participated in the prospective study. The number of samples shipped for resistance testing is added in the last column

KIND OF LABORATORY	GEOGRAPHICAL LOCATION	*M. GENITALIUM* MOLECULAR DETECTION ASSAY	NUMBER OF *M. GENITALIUM* TESTS DURING Q1 2022	NUMBER OF POSITIVE *M. GENITALIUM* SAMPLES DURING Q1 2022	*M. GENITALIUM* POSITIVITY RATIO DURING Q1 2022	SAMPLES SHIPPED FOR RESISTANCE TESTING
**PRIVATE LABORATORY**	Flanders	Abbott - Alinity	1379	47	3.4%	12
**HOSPITAL LABORATORY**	Flanders	Abbott - Alinity	17	1	5.9%	3
**HOSPITAL LABORATORY**	Flanders	Abbott - Alinity	20	0	0.0%	1
**PRIVATE LABORATORY**	Wallonia	Abbott - Alinity	5878	218	3.7%	14
**HOSPITAL LABORATORY**	Wallonia	Diagenode MG/TV	116	12	10.3%	14
**HOSPITAL LABORATORY**	Flanders	Elite - STI PLUS MGB	206	3	1.5%	8
**HOSPITAL LABORATORY**	Flanders	Elite - STI PLUS MGB	267	6	2.2%	10
**HOSPITAL LABORATORY**	Wallonia	Elite - STI PLUS MGB	14	0	0.0%	1
**HOSPITAL LABORATORY**	Wallonia	Elite - STI PLUS MGB	965	13	1.3%	12
**PRIVATE LABORATORY**	Flanders	Elite - STI PLUS MGB	187	9	4.8%	12
**HOSPITAL LABORATORY**	Flanders	Laboratory Developed Test (Taqman Array Card, Lifetech)	1167	63	5.4%	15
**HOSPITAL LABORATORY**	Wallonia	Mikrogen	94	4	4.3%	14
**HOSPITAL LABORATORY**	Wallonia	Neumodx - TV/MG	43	6	14.0%	3
**HOSPITAL LABORATORY**	Flanders	R-biopharm Ridagene STI Mycoplasma	39	1	2.6%	2
**PRIVATE LABORATORY**	Flanders	Roche - Cobas® TV/MG	103	13	12.6%	20
**HOSPITAL LABORATORY**	Wallonia	Seegene - Allplex CT/NG/MG/TV	797	13	1.6%	12
**PRIVATE LABORATORY**	Wallonia	Seegene - Allplex CT/NG/MG/TV	1034	69	6.7%	12
**HOSPITAL LABORATORY**	Brussels	Seegene - Allplex CT/NG/MG/TV	0	0	Not Applicable	12
**HOSPITAL LABORATORY**	Flanders	Seegene - Allplex CT/NG/MG/TV	277	26	9.4%	16
**HOSPITAL LABORATORY**	Flanders	Seegene - Allplex STI Essential Assay	399	13	3.3%	11
**HOSPITAL LABORATORY**	Flanders	Seegene - Allplex STI Essential Assay	206	8	3.9%	4
